# Nurturing child social-emotional development: evaluation of a pre-post and 2-month follow-up uncontrolled pilot training for caregivers and educators

**DOI:** 10.1186/s40814-023-01357-4

**Published:** 2023-08-23

**Authors:** Ruth Speidel, Tracy K. Y. Wong, Redab Al-Janaideh, Tyler Colasante, Tina Malti

**Affiliations:** 1 Centre for Child Development, Mental Health, and Policy, University of Toronto, 3359 Mississauga Rd, Mississauga, ON L5L 1C6 Canada; 2 Department of Psychology, University of Toronto, Mississauga, Canada

**Keywords:** Social-emotional development, Mental health, Caregiver training, Educator training, Children

## Abstract

**Background:**

Social-emotional capacities contribute to children’s mental health by helping them navigate their own and others’ emotional states and forge healthy relationships. Caregivers and educators are critical socialization agents in early and middle childhood, but gaps remain in the systematic integration of social-emotional research into caregiver and educator trainings. The aim of this pilot study was to test the feasibility and preliminary efficacy of a social-emotional training designed to promote caregivers’ and educators’ capacities to support social-emotional development in children ages 3–8 years.

**Methods:**

Fifty adults (*n* = 24 caregivers of children ages 3–8 years, *n* = 26 educators working with children ages 3–8 years) participated in a virtual training over 3 weeks. Participants completed pre-training, post-training, and 2-month follow-up questionnaires evaluating their knowledge of social-emotional concepts, use of training strategies, mental health, and satisfaction with the training. Caregivers also reported children’s social-emotional capacities and mental health.

**Results:**

On average, caregivers and educators completed 83% of the virtual training sessions and reported high satisfaction with the training. Further, preliminary evidence indicated that caregivers’ and educators’ knowledge of social-emotional concepts increased pre- to post-training and was maintained at the 2-month follow-up. Increases in caregivers’ and educators’ knowledge and greater use of training strategies were associated with improvements in children’s social-emotional capacities and caregivers’ and educators’ own mental health.

**Conclusions:**

These pilot results support the feasibility of infusing evidence-based social-emotional content into caregiver and educator training initiatives aimed at nurturing child social-emotional development and mental health. The results inform future evaluation of the short- and long-term benefits of this training with a full-scale randomized controlled trial design.

## Key messages

- *What uncertainties existed regarding the feasibility?*
  - Will the training show high acceptability with caregivers and educators as reflected by high uptake, satisfaction, perceptions of improved learning, and an intent to use strategies from a brief, virtual social-emotional training?
  - Will the training provide preliminary evidence of increasing caregivers’ and educators’ knowledge of theory-based social-emotional concepts and their application of this knowledge through strategies in their care for children?
  - Will the training provide preliminary evidence that it supports child social-emotional and mental health outcomes and caregivers’ and educators’ own mental health outcomes?
- *What are the key feasibility findings?*
  - On average, caregivers and educators completed 83% of training sessions. Further, 95% of caregivers and 100% of educators reported being satisfied with the training, suggesting high uptake and satisfaction.
  - Caregivers’ and educators’ knowledge of social-emotional content increased from pre- to post-training and these increases were maintained at 2-month follow-up. Further, caregivers and educators reported high use of training-based strategies at post-training as their mean scores were between the anchor points of *often* and *very often*.
  - Increases in caregiver and educator knowledge and higher use of training strategies were associated with improvements in key child social-emotional and caregiver and educator mental health outcomes.
- *What are the implications of the feasibility findings for the design of the main study?*
  - Findings provide preliminary support for the feasibility of infusing social-emotional content into training initiatives for caregivers and educators.
  - The preliminary results can be applied to inform future full-scale evaluation of the current training with a randomized controlled trial design.
  - The preliminary results emphasize the importance of evaluating practical use of training strategies alongside knowledge increases when considering potential mechanisms that may explain the effects on this training on child, caregiver, and educator outcomes.

## Background

Across the globe, an estimated 13–20% of children and adolescents experience some form of mental disorder [e.g., attention deficit hyperactivity disorder, anxiety disorder, depressive disorder; [1, 2]. These estimates have jumped during the COVID-19 pandemic, escalating already concerning levels of mental health challenges to crisis levels. For example, pre-pandemic estimates of depression and anxiety in children and adolescents were 1–3 and 7–9%, respectively [1, 2], while recent meta-analytic evidence collected during COVID-19 estimates their prevalence to be as high as 25 and 21%, respectively [3]. Supporting positive mental health outcomes from an early age is a public health priority because an estimated 50% of adult mental health challenges have their onset before age 18 [4]. Early mental health challenges often set the stage for short- and long-term challenges in other domains of functioning, including academic, psychosocial, occupational, and physical health outcomes [5]. Therefore, many researchers in fields spanning developmental psychology, clinical psychology, social work, education, and public health focus on identifying early antecedents of poor and positive mental health to inform mental health promotion initiatives.

One well-founded antecedent and protective domain is social-emotional development. Early social-emotional skills set the foundation for mental health and are susceptible to early socialization practices [6, 7], making them a prime focus for mental health interventions. Further, children’s social and emotional capacities undergo rapid development across early childhood and caregivers (e.g., parents) and early childhood educators (e.g., teachers, service providers, and practitioners in child care and early years centers) shape and cultivate children’s early environments [8], making these ideal contexts for early intervention and prevention efforts targeting social-emotional capacities. In this paper, we evaluate the feasibility of a pilot virtual training designed to bolster caregivers’ and educators’ capacities to support children’s social-emotional capacities and mental health.

### Social-emotional capacities as building blocks of mental health

Social-emotional capacities are the range of skills that enable children to manage their emotions, connect with and care for others, and understand and reflect upon how they relate to others and the world around them [8]. Decades of theory and research situate social-emotional capacities as important precursors and indicators of mental health because early mental health challenges often stem from challenges in the social-emotional domain [e.g., difficulties establishing emotional autonomy and peer relationships; [6, 7]. It is theorized that the process of social-emotional development occurs over time through three core social-emotional components: emotion regulation, other-oriented social-emotional processes (e.g., empathy), and self-oriented social-emotional processes (e.g., self-care) [7, 9]. As children’s social environments grow more complex throughout early and middle childhood, their ability to proficiently navigate their own and others’ emotions in a manner that balances their other-oriented (or interindividual) responsibilities with their self-oriented needs and desires is thought to have implications for their relationships and mental health [e.g., likelihood of disruptive behavior disorders, anxieties and emotional symptoms, and pediatric depression; [6, 7, 9]. Empirical work corroborates this theorizing as links have been established between each core social-emotional component and mental health outcomes, such as lowered risk for internalizing and externalizing challenges and higher propensity for prosociality [5, 10]. Hence, the framework of this pilot training focuses on emotion regulation, other-oriented, and self-oriented processes of social-emotional development.

*Emotion regulation* is the ability to control the occurrence, intensity, and expression of one’s emotions and associated behaviors in order to achieve goals and behave appropriately in one’s environment [11]. Emotion regulation develops rapidly during the early years as it increasingly shifts from an externally guided process (i.e., facilitated by caregivers and other sensitive adults) to an internally guided process (i.e., self-regulation) alongside rapid advancements in cognitive and language development [12]. Emotion regulation is a central social-emotional process because it promotes children’s abilities to cope with challenging emotions and situations across different settings in a flexible manner, and it enables children to engage in their world with greater attention and emotional maturity [13]. Further, emotion regulation is a robust indicator of mental health and adjustment, including greater emotional and behavioral well-being, and emotion regulation challenges are central to different psychopathologies, such as conduct disorders and depression [14]. Thus, supporting children’s emotion regulation can provide a foundation for positive functioning across multiple domains.

*Other-oriented social-emotional processes* reflect how children relate to others [9]. One central other-oriented social-emotional process is empathy, or the ability to feel *with* others [15]. Specifically, empathy occurs when one feels an emotion that is similar to or the same as another’s emotion. Related to empathy, sympathy is the other-oriented capacity to feel concern *for* others in distress, but it does not necessarily require feeling the same emotion as the other [16]. Consistent with past literature in this area, we use empathy as an umbrella term to capture both empathy and sympathy as related processes [15]. Empathy has an emotional component and a cognitive component that develop in infancy and early childhood, respectively [17]. Empathy’s emotional component involves emotion sharing, but does not require understanding the source or cause of others’ emotions. The cognitive component involves the capacity to understand and evaluate others’ perspectives and emotions as distinct from one’s own. When children feel empathy for others, it is theorized to orient them to the needs and desires of others, thereby helping them establish and maintain positive social interactions [18]. Indeed, empathy has been empirically linked to lower aggression, greater prosociality, and more positive relationships, all of which have been linked to, or identified as features of better mental health [19, 20]. Thus, cultivating children’s other-oriented social-emotional capacities is another important step toward nurturing their mental health across multiple domains.

*Self-oriented social-emotional processes* are related to how children perceive themselves in relation to others or how they treat others. One prototypical self-oriented social-emotional process is sadness over wrongdoing (i.e., healthy guilt), or feelings of regret after committing a transgression [15]. Sadness over wrongdoing requires the ability to engage in self-reflection and self-evaluation in relation to ethical standards or rules of behavior [15]. It develops swiftly through the early childhood years and is theorized to help children react in a constructive way when they have committed a transgression, such as by repairing the damage caused [17]. Self-oriented social-emotional skills more generally include children’s ability to understand their own strengths and limitations (an early form of self-reflection), which is thought to facilitate self-care and their personal and interpersonal growth [15]. Thus, self-oriented social-emotional capacities are another important piece of the puzzle comprising healthy social-emotional functioning.

Thus, building children’s core social-emotional capacities is thought to help them form stronger, more meaningful relationships while also supporting their personal understanding and gentleness with their own internal processes and emotional challenges (i.e., their capacity to help themselves in the service of helping others) [21, 22]. Adopting this perspective, the current pilot training aimed to improve caregivers’ and educators’ research-based knowledge of social-emotional development while also supporting their ability to nurture social-emotional capacities and mental health in children and themselves.

### The role of caregivers and educators in children’s social-emotional development

Social-emotional capacities have biological and genetic components and are also subject to early environmental influences. From an attachment theory perspective, the sensitivity of early caregiver–child relationships shapes children’s working scripts of relationships and their understanding of appropriate and inappropriate cognitions, behaviors, and motivations for behavior [23]. Warmth, sensitivity, and structure in early caregiver–child relationships have been positively linked with children’s emotion regulation, empathy, and mental health [24, 25]. Beyond the early caregiver–child relationship, ecological systems theory and relational-developmental systems of care approaches argue that children develop in a nested web of environments—the home, school, broader community, and beyond—all of which interweave and bring in outside actors that influence child development [26, 27]. For example, positive educator–child interactions and school climate have been linked with greater child social-emotional capacities and reduced problem behaviors [28]. Thus, targeting change at multiple levels of children’s environmental systems through caregivers and educators may increase the breadth of training impacts on children’s social-emotional development and mental health.

### Existing approaches to support children’s social-emotional capacities

Given the established links between social-emotional capacities and positive mental health and given caregivers’ and educators’ roles in nurturing these capacities, prevention and intervention efforts often target social-emotional capacities as an avenue for supporting child mental health [29]. Such initiatives—often called *social and emotional learning* (SEL) programs—have been shown to effectively promote children’s mental health and prosociality, even several years after program implementation and especially when they are implemented in early childhood [for meta-analytic evidence, see [30–32].

There have been sweeping efforts to incorporate social-emotional content into school curricula in efforts to support children’s social-emotional development [33]. For example, by 2020, all 50 US states had adopted preschool SEL learning standards and 18 states had K–12 SEL learning standards [34]—a pattern of social-emotional educational reform that has repeated globally [33]. Despite these progressions, gaps remain in the effective integration of social-emotional content into educational settings. First, there are limitations in the application of cutting-edge social-emotional theory and research into practice [9, 35]. There is a notable dearth of social-emotional content in educators’ professional development opportunities [33]. For example, a scan of 3916 courses in US college education programs revealed that only 6% included content on emotion regulation or self-management, 2% included content on other-oriented social-emotional processes, and 1% included content on self-oriented social-emotional processes [36]. Corroborating this gap, educators report receiving limited training and limited confidence in their ability to support SEL in a sustainable manner [37, 38]. This trend is concerning given the above-noted increases in mandated SEL curricular components. Similar limitations are present—if not more exacerbated—in early childhood education settings outside of schools, such as in child care and service settings where professional qualifications and curricula tend to vary more widely [39]. Thus, supporting a diverse array of early childhood educators with research-based knowledge and application of social-emotional capacities is a logical first step to reducing the most pressing research–practice gaps in this area.

There are even fewer training opportunities for caregivers. However, early parenting interventions have been shown to improve parenting factors, such as attachment, parental warmth, sensitivity, and positive discipline, which in turn support child social-emotional development and mental health [40–42]. There are growing calls to include caregivers in efforts to support child social-emotional development [42] and recommendations for scaling up SEL programming often advocate for caregiver components so that social-emotional improvements in early learning environments, such as daycares and schools, can be supplemented and extended by social-emotional support at home [43].

Finally, growing evidence suggests that it may not be enough to increase caregivers’ and educators’ knowledge of children’s social-emotional development; rather, supporting caregivers’ and educators’ own capacities—especially their mental health—can improve the efficacy of provider-focused efforts to support child development [44]. From this perspective, social-emotional training initiatives may benefit from supporting caregivers’ and educators’ own emotion regulation, other-oriented social-emotional processes, self-oriented social-emotional processes, and mental health. Current SEL promotion initiatives in schools are often limited in scope and fail to incorporate broader systems-level practices that nurture all parties involved in children’s caring environments [45]. This gap is particularly stark for caregivers and educators, as these providers are often balancing their own and others’ needs in high-stress environments, which can contribute to mental health challenges in themselves that spillover into challenges in providing appropriate care for children [46, 47]. Further, in the context of COVID-19, early childhood educators are at elevated risk for compassion fatigue, secondary traumatic stress, and burnout [48], which can negatively impact their ability to provide sensitive care. Hence, expert recommendations in the wake of COVID-19 advocate for initiatives aimed at reducing caregiver and educator stress and supporting mental health and self-compassion [49].

### The present training

This pilot study integrates social-emotional research into a brief virtual training for caregivers and educators of children ages 3 to 8. The goal of the pilot study was to evaluate the feasibility of a brief, virtual training approach, with the ultimate aim of informing the future implementation of a large-scale training within a randomized controlled trial design that aims to increase caregivers’ and educators’ knowledge of children’s social-emotional development, positive early relationships, and coping with stress, as well as their capacity to support their own and children’s social-emotional development and mental health. The pedagogical approach for the training focuses on *knowledge*, *practice*, and *routine* to align with three core principles of adult learning: (1) Caregiver and educators have existing competencies that can be bolstered with research-based *knowledge* [50]; (2) Learning is an active and collaborative *practice* (rather than passive and isolated [51]; and (3) Learning is continuous and is flexibly reshaped through *routine* and reflective practice [52]. The knowledge component of our pedagogical approach cuts across all elements of the training by supporting caregivers and educators with evidence-based clinical–developmental knowledge of social-emotional development that builds upon experiences and existing competencies. The practice component is supported through the use of hands-on opportunities to apply concepts and share lived experiences in an interactive setting. Finally, the routine component is nurtured through the provision of continued learning opportunities designed to offer continued support and to understand strengths and barriers to sustained learning (see “Methods” for detailed information on the training delivery and content).

### Research questions

To evaluate feasibility in the present proof-of-concept study, our research questions were the following: (1) Will the training show high acceptability, with caregivers and educators showing high uptake (i.e., retention), satisfaction, perceptions of enhanced learning, and intent to use strategies from the brief, virtual social-emotional training? (2) Will preliminary evidence suggest that caregivers and educators increase in their knowledge of core social-emotional concepts, and will they report high use of training strategies? *3) Will increases in caregiver knowledge and higher use of training strategies by caregivers be associated with increases in their children’s social-emotional capacities and mental health? (4) Will increases in caregivers’ and educators’ knowledge and their higher use of training strategies be associated with improvements in their own mental health?

## Methods

### Participants

**Fig. 1:**
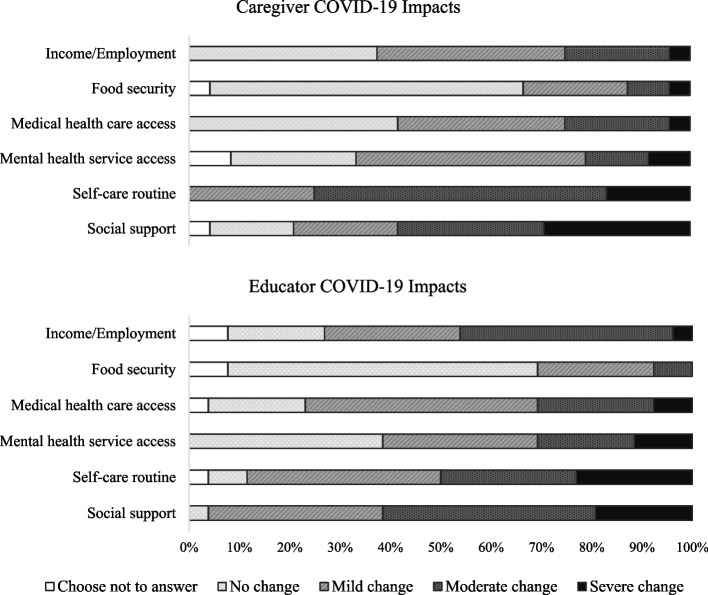
Caregiver and Educator COVID-19 impacts. Full scale: 0 = *no negative change*, 1 = *mild negative change*, 2 = *moderate negative change*, 3 = *severe negative change*. There were no statistically significant differences in caregivers’ and educators’ mean levels (ps > .11)

Fifty caregivers (*n* = 24) and early childhood educators (*n* = 26) participated in the training. Caregivers and educators were recruited through various early years and child care sector organizations who served as community partners on the current project. Specifically, our partners disseminated flyers and social-media posts and shared the opportunity via word of mouth at their programming sites in [masked for review] region in [masked for review], Canada. We determined the planned sample size to be in line with past similar non-randomized feasibility studies [53, 54]. Eligibility criteria for caregivers included being the primary caregiver of at least one child aged 3–8 years and living in [masked for review]. Eligibility criteria for educators included working with children 3–8 years in the education, early years programming, or child care sectors of [masked for review]. Educators’ years of experience ranged from less than 4 to 32 (*M* = 15.31, *SD* = 8.36). The participating educators worked in a variety of settings, including in schools as teachers (42.3%), in child care centers as child care providers (42.3%), and in early years child and family programming centers and resource support organizations as early childhood educators (15.4%). The sample’s demographics are reported in Table 1. Aligned with the high rates of racial and ethnic diversity in this region, 83% of caregivers and 62% of educators identified as a member of a visible minority group. Among caregivers who migrated to Canada, 71% reported arriving in Canada between 2 and 35 years before study enrollment (*M* = 12.8, *SD* = 9.6), with 82% reporting that their child was born in Canada. The current study occurred in October 2021, in the midst of the COVID-19 pandemic, approximately 18 months after the first COVID-19 cases and lockdowns in Ontario, and while the Delta variant was considered the key variant of concern. At the start of the study, a lockdown was not in effect and approximately 71.5% of Ontarians had been vaccinated with two doses [55]. To provide relevant context, caregivers and educators reported COVID-19 impacts they had experienced across various areas of their lives, including negative changes in their access to social supports, self-care routine, emotional health and well-being service access, medical health care access, food security, and income/employment (see Fig. 1).

**Table 1:** Caregiver and educator demographics

	**Caregiver (*n* = 24)**		**Educator (*n* = 26)**	
**Variable**	***M***	(***SD***)	***M***	(***SD***)
Caregiver/educator age	37.82	(3.43)	43.79	(10.91)
Child age	5.11	(1.65)	–	–
**Variable**	***n***	(***%***)	***n***	(***%***)
Caregiver/educator gender				
Man	2	(8.3%)	0	(0.0%)
Woman	22	(91.7%)	26	(100%)
Child gender				
Boy	13	(54.2%)	–	–
Girl	11	(45.8%)	–	–
Migration status				
Migrated to Canada from another country	17	(70.8%)	–	–
Did not migrate to Canada	7	(29.2%)	–	–
Population group				
Choose not to answer	–	–	1	(3.8%)
Black	2	(8.3%)	3	(11.5%)
Chinese	5	(20.8%)	0	(0.0%)
Filipino	1	(4.2%)	0	(0.0%)
Indigenous	1	(4.2%)	0	(0.0%)
Latin American	1	(4.2%)	2	(7.7%)
South Asian	9	(37.5%)	10	(38.5%)
Southeast Asian	1	(4.2%)	0	(0.0%)
West Asian	1	(4.2%)	0	(0.0%)
White	4	(16.7%)	10	(38.5%)
Education				
Choose not to answer	1	(4.2%)	1	(3.8%)
High school equivalent	1	(4.2%)	0	(0.0%)
College, CEGEP, or non-university diploma	3	(12.5%)	12	(46.2%)
University diploma or degree	19	(79.2%)	13	(50.0%)
Employment				
Choose not to answer	1	(4.2%)	1	(3.8%)
Parental Leave	1	(4.2%)	0	(0.0%)
Unemployed	6	(25.0%)	0	(0.0%)
Part time	1	(4.2%)	5	(19.2%)
Full time	15	(62.5%)	20	(76.9%)
Income				
Choose not to answer	8	(33.3%)	8	(30.8%)
$20 k–$49,999 CAD	2	(8.3%)	2	(7.7%)
$50 k–$99,999 CAD	5	(20.8%)	9	(34.6%)
$100,000 CAD or more	9	(37.5%)	7	(26.9%)
Marital status				
Choose not to answer	1	(4.2%)	1	(3.8%)
Married	21	(87.5%)	17	(65.4%)
Not married	2	(8.3%)	8	(30.8%)

### Procedure

The study was conducted according to the guidelines of the Declaration of Helsinki and was approved by the [masked for review] Research Ethics Board [protocol number masked for review]. Caregivers and educators provided informed consent prior to participating. The training was delivered virtually in October 2021 over the course of 1 month via a web-based learning management system and was facilitated by two developmental psychology PhDs and an educator with experience in family and service provider supports. To facilitate training evaluation, caregivers and educators completed a pre-survey, a series of post-surveys (after each module session), and a 2-month follow-up survey.

### The RAISE training

The training, formally referred to as *R*esearch and Practice Partnership: Building *A*wareness and *I*ncreasing *S*ocial-Emotional Capacity in the *E*arly Years (RAISE), is comprised of three modules, with each module delivered weekly over the course of 3 weeks. Module 1 introduces the three core components of social-emotional development outlined in the introduction of this paper: emotion regulation, other-oriented social-emotional capacities, and self-oriented social-emotional capacities [9]. The module presents the definitions, developmental importance, and typical development of each social-emotional component from ages 3–8 years, and provides practical strategies that caregivers and educators can use to promote each component, such as emotion coaching, mindfulness, and modelling. Module 2 highlights the importance of early relationships for supporting healthy child social-emotional development, based in relational-developmental systems approaches of care [26] with a focus on knowledge and practical application of three core caregiving processes grounded in developmental theory and cross-cultural empirical work: warmth, sensitivity, and structure/autonomy support [56, 57]. Module 3 highlights mental health, including the impacts of stress on social-emotional development and resilience, and emphasizes protective factors that can support children’s, caregivers’, and educators’ mental health during and after stressful situations, such as COVID-19 [58]. Further, this module includes an additional section for educators on the utility of assessment tools to help with monitoring, evaluating, and understanding individual children’s social-emotional and stress-management capacities.

Each module included an asynchronous solo session and a synchronous group session. Each asynchronous session consisted of three 15-min videos that participants viewed at their own pace during the week. The videos delivered the core knowledge-based training content and were accompanied by at-home and at-care strategies for participants to practice during the week. The application-based synchronous group sessions occurred at the end of each week were 90 min long, and included 3 components: (1) a guided mindfulness exercise, (2) a semi-structured question-and-answer group discussion and reflection, and (3) interactive small- and large-group practice activities. Thus, the training adopted a flipped classroom learning approach, whereby participants engaged in self-guided learning in combination with interactive learning activities designed to build upon skills and knowledge of basic concepts by making connections between personal experiences and others’ experiences. This flexible and interactive blended approach supports improved learning relative to more traditional teaching methods [59]. Two synchronous group sessions were run each week (1 session for caregivers, *n* = 24; 1 session for educators, *n* = 26). Each synchronous session was facilitated by two developmental psychologists and a family supports practitioner. Synchronous sessions supported active participant involvement in two central ways: (1) multiple avenues for participant sharing were provided for different preferences (i.e., verbally or orally, via the chat feature, or anonymously through polls and online tools used throughout the sessions), (2) we made use of a combination of large and small group activities (with 3–4 participants in the small group activities) to give all participants the opportunity to share and learn from each other. Table 2 provides more details on the RAISE training, including information about the aim, content, and strategies included in each session.

**Table 2:** RAISE training description

Module	Session	Length	Aim	Content	Strategies
1	Asynchronous	45 min	Build knowledge about 3 core components of social-emotional development (the 3Es: emotion regulation, empathy for the self [self-oriented emotion], empathy for others[other-oriented emotion]), and strategies to support The 3Es	• Definitions of each social-emotional component • Developmental importance of each component • Typical development of each component • Strategies to support each component	Emotion Regulation: • Being an emotion coach • Practicing mindfulness • Modelling emotion regulation Empathy for Others: • Promoting perspective taking • Encouraging and noticing small acts of kindness • Modelling empathy Empathy for the Self: • Encouraging self-awareness • Actively linking child actions to consequences • Modelling self-kindness
	Synchronous	90 min	Practice applying knowledge to support the 3Es	• Mindfulness activity • Discussion of asynchronous session and reflection • Activities and practice	
2	Asynchronous	45 min	Build knowledge about 3 core components of early relationships (the 3Cs: connecting, caring, coaching), and strategies to engage in the 3Cs	• Definitions of each early relationship component • Developmental importance of each component • Significance for social-emotional development • Strategies to engage in each component	Connecting: • Observing and being mindful of children • Creating times of joy • Practicing self-kindness (i.e., taking time for you) Caring: • Recognizing and attuning to children’s unique cues • Thinking about what children are trying to communicate with their behaviors • Responding sensitively Coaching: • Setting clear, consistent, and fair expectations • Using routines • Providing children choice within limits
	Synchronous	90 min	Practice applying knowledge to support the 3Cs	• Mindfulness activity • Discussion of asynchronous session and reflection • Activities and practice	
3	Asynchronous	45 min	Build knowledge about growth and well-being in children and adults, and strategies to support well-being in contexts of stress and adversity	• Maslow’s hierarchy of needs • Positive psychology • Stress • Resilience	Supporting children in times of stress: • Making time for joy • Making sleep a priority • Taking a step in children’s shoes Supporting ourselves in times of stress: • Acknowledging negative thoughts • Changing thought patterns (cognitive reappraisal) • Finding humor
	Synchronous	90 min	Practice applying knowledge to support well-being in children and caregivers	• Mindfulness activity • Discussion of asynchronous session and reflection • Activities and practice	

The training focuses on early to middle childhood—specifically the 3–8 age range—for two reasons. First, the core social-emotional components that are the focus of the current training undergo significant development during this period, making these optimal ages to benefit from the training. For example, around age 3, emotion regulation, sadness over wrongdoing, problem-solving, perspective taking, and empathy clearly emerge and rapidly develop alongside increases in the complexity of children’s social environments as they navigate different childcare and educational settings [7]. Second, caregivers and educators serve as critical socialization agents during this time, and children are particularly moldable to socialization efforts during these periods [60]. Given that different elements of these core capacities develop at different rates during the 3–8 age range, developmentally appropriate strategies were tailored for younger (3–5 years) and older (6–8 years) children to ensure that the strategies were appropriate to each age range. For example, one of the strategies designed to support emotion regulation was to be an emotion coach by engaging in emotionally supportive and validating emotion-based discussions with children. The training provides examples of how these conversations may look for younger children, with the caregiver/educator playing a more active role in elaborating and providing labels for children’s emotions (e.g., “You look like you are sad”), versus older children, where the caregiver/educator might encourage children to lead the conversation and label how they are feeling (e.g., “How are you feeling?”).

### Measures

**Fig. 2:**
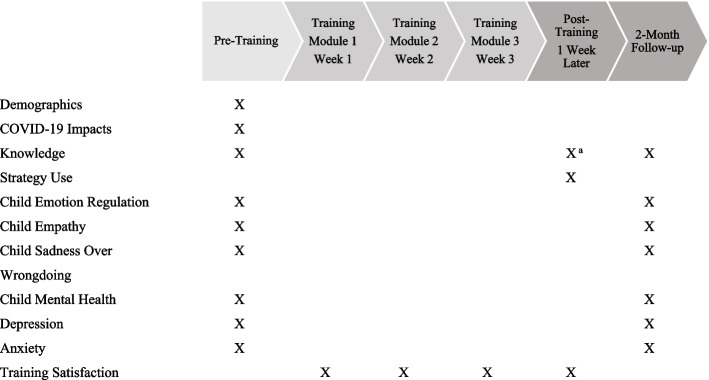
Training schedule and measurement timing for the current study. ^a^ Applicable knowledge items were assessed after each module session and were averaged together to form one post-training knowledge variable

Figure 2 details the training schedule and when specific measures were assessed.

#### COVID-19 impacts

At pre-training, caregivers and educators reported on six items assessing negative changes due to the pandemic across various areas of their lives. Items were adapted from the Coronavirus Impact Scale [61] and included negative changes in access to social supports, mental health services, medical health care, food security, and income/employment. An additional item assessing changes in self-care practices was developed in collaboration with our community partners. The items were rated on a 4-point scale (1: *no change*, 2: *mild change*, 3: *moderate change*, 4: *severe change*) and included example anchors to further clarify the extent of negative change. For example, the social supports item was: *How much has the Coronavirus pandemic impacted your access to extended family and non-family social supports*. The response options for this item were: *No change, Mild change–Continued visits with social distancing and/or regular phone calls and/or tele-video or social media contacts, Moderate change–Loss of in person and remote contact with a few people, but not all supports, Severe change–Loss of in person and remote contact with almost all or all supports*.

#### Satisfaction with the training

After each asynchronous and synchronous session, caregivers and educators reported their satisfaction with the session on a 10-point scale (1: *very dissatisfied*, 4: *somewhat dissatisfied*, 7: *somewhat satisfied*, 10: *very satisfied*). One week after training completion, participants reported their overall satisfaction with the training.

#### Perceptions of learning

After each asynchronous and synchronous session, caregivers and educators reported their perceptions of their own learning from the session on one item, “To what extent do you feel that you learned something new?”. The item was rated on a 10-point scale (1: *not at all*, 4: *to a small extent*, 7: *to a moderate extent*, 10: *to a large extent*).

#### Intent to use strategies

After each asynchronous and synchronous session, caregivers and educators reported the extent to which they felt they were likely to use any of the strategies described in the session on a single item, “To what extent would you use any of the strategies mentioned in this session?”. The item was rated on a 10-point scale (1: *not at all*, 4: *to a small extent*, 7: *to a moderate extent*, 10: *to a large extent*).

#### Use of training strategies

One week after the training, caregivers and educators reported their actual use of training-based strategies (e.g., mindfulness, emotion coaching) on a single item, “To what extent do you use any of the strategies from the training?”. The item was rated on a 10-point scale (1: *not at all*, 4: *sometimes*, 7: *often*, 10: *very often*).

#### Knowledge of training content

At pre-training, after each asynchronous and synchronous session, and at the 2-month follow-up, caregivers and educators rated their understanding of core social-emotional concepts targeted in the training using 18 items. The items were developed by the first, fourth, and fifth authors to assess knowledge of core social-emotional concepts targeted in the training (e.g., “I understand what emotion regulation is and how it develops”), and knowledge of how to apply core social-emotional concepts (e.g., “I know how to support children’s emotion regulation”). Items were rated on a 10-point scale (1: *entirely disagree*, 4: *somewhat disagree*, 7: *somewhat agree*, 10: *entirely agree*). Individual scores across the 18 items were averaged to form pre-training, post-training,^1^ and 2-month follow-up scores (*α*_caregiver_ = 0.95 and 0.99; *α*_educator_ = 0.96 and 0.98).

#### Child social-emotional capacities

At pre-training and the 2-month follow-up, caregivers reported their child’s emotion regulation, empathy for others, and sadness over wrongdoing using the Social-Emotional Responding Task [62]. These capacities were assessed as they map onto the core components of social-emotional development highlighted in the training: emotion regulation, other-oriented social-emotional capacities, and self-oriented social-emotional capacities, respectively. Caregivers completed 12 items assessing emotion regulation (e.g., “My child calmly deals with what is making them mad”, *α* = 0.89 and 0.91), 4 items assessing empathy (e.g., “My child feels bad for other children who are sad”, *α* = 0.70 and 0.92), and 4 items assessing sadness over wrongdoing (e.g., “When my child does something that makes another child feel sad, they feel sad”, *α* = 0.76 and 0.77). Items were rated on a 4-point scale (0: *not at all*, 1: *sometimes true*, 2: *often true*, 3: *almost always true*) and were averaged to form emotion regulation, empathy, and sadness over wrongdoing scores.

#### Child mental health challenges

At pre-training and the 2-month follow-up, caregivers rated 7 items assessing their child’s internalizing symptoms (e.g., “My child has many worries or often seems worried”; *α* = 0.78 and 0.81) and 7 items assessing their child’s externalizing symptoms (e.g., “My child often fights with other children or bullies them”; *α* = 0.69 and 0.78) from the Strengths and Difficulties Questionnaire [63].^2^ Items were rated on a 3-point scale (0: *not true*, 1: *somewhat true*, 2: *certainly true*) and averaged into a single mental health challenges variable, with higher scores indicating greater mental health challenges.

#### Caregiver and educator depressive and anxiety symptoms

At pre-training and the 2-month follow-up, caregivers and educators reported their depressive symptoms over the previous 2 weeks with 2 items from the Patient Health Questionnaire-2 [64] (e.g., “Little interest or pleasure in doing things”; Pearson’s *r*_caregiver_ = 0.76–0.82; *r*_educator_ = 0.48 and 0.74) and their anxiety symptoms over the same period with 2 items from the Generalized Anxiety Disorder-2 [65] (e.g., “Not being able to stop or control worrying”; *r*_caregiver_ = 0.77 and 0.84; *r*_educator_ = 0.64 and 0.84). Items were rated on a 4-point scale (0: *not at all*, 1: *several days*, 2: *over half the days*, 3: *nearly every day*) and averaged into respective depressive and anxiety symptoms scores.

### Data analytic plan

For our first research question of interest, we assessed retention throughout the training via course metrics available through the web-based LMS (learning management system), including the percent of registered learners who completed each session. Consistent with conventions for effective interventions, we considered a retention rate at or above 80% to be a marker of adequate retention [66]. Further, we conducted a descriptive analysis of caregivers’ and educators’ training satisfaction based on their self-reports of satisfaction with the training.

To assess our second research question of if this pilot showed preliminary evidence that caregivers’ and educators’ knowledge of social-emotional concepts increased between pre-training, post-training, and the 2-month follow-up, we conducted a repeated-measures ANOVA in SPSS 28 evaluating changes in knowledge across the three timepoints. Group status (0: *caregiver*, 1: *educator*) was considered as a between-subjects factor to evaluate differences between caregivers and educators in changes in knowledge over time. If Mauchly’s test of sphericity was statistically significant, suggesting that sphericity could not be assumed, the Greenhouse–Geisser results were reported. Additionally, where significant univariate effects emerged, follow-up pairwise comparisons were conducted with Bonferroni corrections. To evaluate the prevalence of training strategy use at post-training, caregivers’ and educators’ use of training strategies was evaluated using a descriptive analysis of means, standard deviations, and ranges.

To answer our third research question of whether there was preliminary evidence that changes in caregivers’ knowledge and caregivers’ use of training strategies were associated with changes in child social-emotional and mental health outcomes, we conducted four multiple regression analyses in M*plus* 8 [67], one for each child outcome. The independent variables in each model were changes in caregiver knowledge from pre- to post-training and caregiver use of strategies at post-training. We also investigated the interaction of these two variables because we expected that increases in knowledge may not have a downstream impact on child functioning unless the knowledge was actually put into practice. The dependent variable in each model was changes in the child outcome (emotion regulation, empathy for others, sadness over wrongdoing, mental health challenges) from pre-training to the 2-month follow-up. Specifically, we formed difference score variables reflecting changes in caregiver knowledge by subtracting knowledge at pre-training from knowledge at post-training such that positive values on the change score indicated increases in knowledge and negative scores indicated decreases in knowledge from pre- to post-training. Similarly, we formed change scores for each child outcome by subtracting the 2-month follow-up score from the pre-training score. All regression models were designed to maximize a temporal sequence from the independent variables to the dependent variables (i.e., such that changes in knowledge were considered from pre- to post-training, use of training-based strategies was considered at post-training, and changes in each outcome variable of interest were considered from pre-training to the 2-month follow-up).

To answer our final research question about whether there was preliminary evidence that changes in caregivers’ and educators’ knowledge and their higher use of training strategies were associated with changes in their depressive and anxiety symptoms, we conducted two multiple regression analyses in M*plus* 8 (one for each of depressive and anxiety symptoms). In each regression, the independent variables were changes in caregiver and educator knowledge from pre- to post-training, caregiver and educator use of strategies at post-training, and the interaction between these two variables. In one model, the dependent variable was changes in caregiver and educator depressive symptoms from pre-training to the 2-month follow-up. In the other model, the dependent variable was changes in anxiety symptoms from pre-training to the 2-month follow-up.

### Missing data analysis

To evaluate patterns of missingness in the data, Little’s test of missing completely at random (MCAR) was conducted for the primary study variables of each research question. Little’s test was nonsignificant for research question 2, *χ*^2^(10) = 13.78, *p* = 0.18, and research question 3, *χ*^2^(35) = 47.60, *p* = 0.08, suggesting that the missing data did not violate assumptions of MCAR. Little’s test was statistically significant for research question 4, *χ*^2^(45) = 75.1, *p* = 0.003, suggesting that the missing data violated the assumption of MCAR. Specifically, caregivers and educators who reported higher depressive and anxiety symptoms at pre-training and the 2-month follow-up were more likely to have missing data (*p*s =  < 001–0.03). Thus, to handle missing data, research question two was conducted in SPSS 28 using multiple imputation with the expectation–maximization method, with demographic and primary study variables included as auxiliary variables over 25 iterations, as this approach is robust under assumptions of MCAR. Research questions 3 and 4 were evaluated in M*plus* 8 using full information maximum likelihood (FIML) estimation as this method is robust under conditions of MAR and MCAR. To maximize power, all multiple regression models were tested using 5000 bootstrap resamples and the bootstrapped 95% confidence interval results were assessed to determine significance.

## Results

Means and standard deviations are reported in Table 3. Below, we report the results for each of our objectives.

**Table 3:** Means and standard deviations of primary study variables

	**Caregiver**				**Educator**				**Full sample**			
	*M*	*SD*	min	max	*M*	*SD*	min	max	*M*	*SD*	min	max
Knowledge (Pre)	6.92	(1.49)	3.72	8.59	8.56	(1.05)	5.33	10.00	7.79	(1.51)	3.72	10.00
Knowledge (Post)	8.48	(1.02)	6.50	10.00	9.26	(0.64)	7.78	10.00	8.90	(0.91)	6.50	10.00
Knowledge (2-Mo)	8.67	(0.82)	7.06	10.00	9.28	(0.64)	8.00	10.00	8.99	(0.79)	7.06	10.00
Use of Strategies (Post)	8.17	(1.69)	5.00	10.00	8.17	(1.69)	4.00	10.00	8.17	(1.66)	4.00	10.00
Depression (Pre)	0.80	(0.88)	0.00	3.00	0.81	(0.84)	0.00	2.50	0.80	(0.85)	0.00	3.00
Depression (2-Mo)	0.70	(0.75)	0.00	2.50	0.87	(0.91)	0.00	3.00	0.79	(0.83)	0.00	3.00
Anxiety (Pre)	1.02	(0.99)	0.00	3.00	1.00	(0.94)	0.00	3.00	1.01	(0.95)	0.00	3.00
Anxiety (2-Mo)	0.93	(0.74)	0.00	3.00	0.96	(0.87)	0.00	3.00	0.95	(0.80)	0.00	3.00
Child Emotion Regulation (Pre)	0.84	(0.53)	0.00	1.75	–	–	–	–	–	–	–	–
Child Emotion Regulation (2-Mo)	0.88	(0.50)	0.00	1.83	–	–	–	–	–	–	–	–
Child Empathy (Pre)	1.92	(0.61)	0.75	3.00	–	–	–	–	–	–	–	–
Child Empathy (2-Mo)	2.17	(0.80)	0.25	3.00	–	–	–	–	–	–	–	–
Child Sadness Over Wrongdoing (Pre)	1.56	(0.66)	0.25	3.00	–	–	–	–	–	–	–	–
Child Sadness Over Wrongdoing (2-Mo)	1.77	(0.62)	0.00	2.50	–	–	–	–	–	–	–	–
Child Mental Health Challenges (Pre)	0.84	(0.38)	0.29	1.57	–	–	–	–	–	–	–	–
Child Mental Health Challenges (2-Mo)	0.75	(0.36)	0.07	1.57	–	–	–	–	–	–	–	–

The actual allowable ranges on each variable were as follows: Knowledge (0–10), Use of Strategies (1–10), Depression (0–3), Anxiety (0–3), Child Emotion Regulation (0–3), Child Empathy (0–3), Child Sadness over Wrongdoing (0–3), and Child Mental Health Challenges (0–2)

### Research question 1

One caregiver who initially accepted did not start the training, leaving a sample of 24 caregivers. All 26 educators who accepted started the training. Of the 50 participants, 45 (90%) completed the module 1 asynchronous session, 43 (86%) completed the module 1 synchronous group session, 38 (76%) completed the module 2 asynchronous session, 42 (84%) completed the module 2 synchronous group session, 39 (78%) completed the module 3 asynchronous session, and 41 (82%) completed the module 3 synchronous group session. On average, participants completed 83% of the training sessions and rates of attendance were similar for caregivers and educators, *t*(48) =  − 1.10, *p* = 0.28. Ninety-six percent of caregivers reported being somewhat satisfied (i.e., 7 out of 10 on our scale) to very satisfied (i.e., 10 out of 10) after each training session (with one-third [33%] reporting being very satisfied). At post-training, 95% of caregivers reported being somewhat satisfied to very satisfied with the training overall (with one-third [33%] reporting being very satisfied). One hundred percent of educators reported being somewhat satisfied to very satisfied after each session (with over half [60%] reporting being very satisfied). At post-training, 100% of educators reported being somewhat satisfied to very satisfied with the training overall (with over half [56%] reporting being very satisfied).

Regarding suitability and intent to use the training, 95% of caregivers reported that they felt they had learned something new in each training session to a moderate to large extent (with 30% reporting they felt they learned something new to a large extent). Ninety-seven percent of caregivers reported that they were likely to try the strategies mentioned in each session to a moderate to large extent (with 47% reporting they were likely to try the strategies to a large extent). Ninety-six percent of educators reported that they felt they had learned something new in each training session to a moderate to large extent (with 44% reporting they felt they learned something new to a large extent). One hundred percent of educators reported that they were likely to try the strategies mentioned in the session to a moderate to large extent (with 55% reporting they were likely to try the strategies to a large extent).

### Research question 2

The test of within-subjects effects revealed statistically significant changes in participant knowledge between at least two time points, *F*(1.51, 72.67) = 46.47, *p* < 0.001, ${\upeta }_{p}^{2}$ = 0.49. Specifically, across participants, there were significant increases in knowledge from pre- to post-training (mean difference = 1.13, *SE* = 0.16, *p* < 0.001; *d* = 0.90), and from pre-training to the 2-month follow-up (mean difference = 1.21, *SE* = 0.16, *p* < 0.001; *d* = 0.90). There was no change in knowledge from post-training to the 2-month follow-up (mean difference = 0.08, *SE* = 0.09, *p* = 1.00; *d* = 0.10). The pattern of results suggested that caregivers’ and educators’ knowledge increased from pre- to post-training and that these increases were maintained by the 2-month follow-up.

**Fig. 3:**
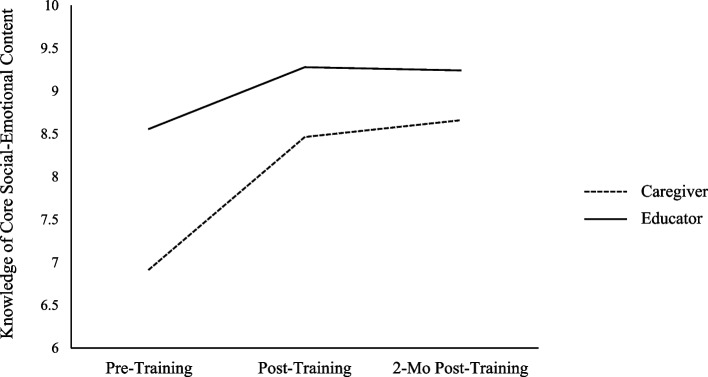
Caregiver and educator knowledge at pre-training, post-training, and the 2-month follow-up. Differences between pre- and post-training were significant for both caregivers and educators and levels were maintained 2 months later

The test of within-subjects by between-subjects interaction revealed significant differences in knowledge between caregivers and educators, *F*(1.51, 72.67) = 7.82, *p* = 0.002, ${\upeta }_{p}^{2}$ = 0.14. To probe the interaction, follow-up pairwise comparisons were conducted and indicated that educators were higher in knowledge relative to caregivers at pre-training (mean difference = 1.64, *SE* = 0.36, *p* < 0.001; *d* = 1.30), post-training (mean difference = 0.82, *SE* = 0.23, *p* < 0.001;* d* = 1.02), and the 2-month follow-up (mean difference = 0.58, *SE* = 0.21, *p* = 0.008; *d* = 0.79). In other words, educators started at a higher baseline level of knowledge of social-emotional concepts relative to caregivers, and maintained this gap across the training. To further probe the interaction effect, follow-up pairwise comparisons were conducted to examine patterns of change in educators and caregivers separately. These follow-up comparisons revealed that although the general patterns of change in knowledge were consistent between caregivers and educators (see Fig. 3), the rate of change was higher for caregivers relative to educators. Specifically, both groups increased in knowledge from pre- to post-training (*ps* < 0.001–0.006), but the effect size was large for caregivers (mean difference = 1.55, *SE* = 0.23, *p* < 0.001; *d* = 1.23) and medium for educators (mean difference = 0.72, *SE* = 0.22, *p* = 0.006; *d* = 0.57). Similarly, both groups increased in knowledge from pre-training to the 2-month follow-up (*ps* < 0.001–0.01), but the effect size was large for caregivers (mean difference = 1.74, *SE* = 0.23, *p* < 0.001; *d* = 1.38) and medium for educators (mean difference = 0.68, *SE* = 0.22, *p* = 0.01; *d* = 0.54). Both groups remained relatively consistent in their change in knowledge from post-training to the 2-month follow-up (*ps* = 0.45–1.00; *d*_caregiver_ = 0.25, *d*_educator_ = 0.05). Thus, the significant within-subjects by between-subjects interaction effect was driven by caregivers (who were lower in knowledge relative to educators throughout the training) showing slightly steeper improvements in their knowledge from pre- to post-training and from pre- to 2-month follow-up, relative to educators.

On average, participants reported high strategy use at post-training (*M*_caregiver_ = 8.17, *SD*_caregiver_ = 1.69, Range_caregiver_ = 5–10; *M*_educator_ = 8.17, *SD*_educator_ = 1.69, Range_educator_ = 4–10), as their mean scores were between the anchor points of *often* (7) and *very often* (10). One hundred percent of participants reported using the training-based strategies at least *sometimes* (4).

### Research question 3

**Fig. 4:**
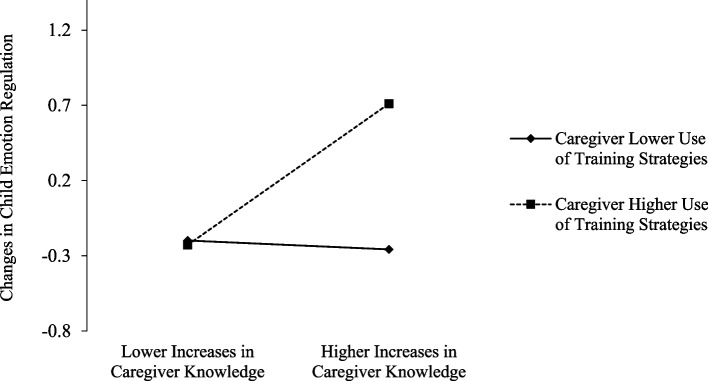
Interaction plot. Increases in knowledge from pre- to post-training in relation to changes in child emotion regulation from pre- to 2-month post-training at lower (− 1 SD) and higher (+ 1 SD) levels of use of training strategies post-training. The observed range of changes in child emotion regulation was − 0.75 to 1.08. Positive scores on changes in emotion regulation denote increases, whereas negative scores on changes in emotion regulation denote decreases

As per Table 4, significant main effects of changes in caregiver knowledge from pre- to post-training and caregiver training-based strategy use at post-training on changes in child emotion regulation from pre-training to the 2-month follow-up, *b* = 0.55, 95% CI [0.003, 0.976] and *b* = 0.60, 95% CI [0.154, 0.893], respectively, were qualified by their significant interaction, *b* = 0.69, 95% CI [0.039, 1.166]. Specifically, when caregivers’ use of strategies was higher, there was a positive association between increases in caregiver knowledge and increases in child emotion regulation, *b* = 1.18, 95% CI [0.106, 2.047]. There was no such association when use of strategies was lower, *b* =  − 0.07, 95% CI [− 0.386, 0.196] (see Fig. 4).

**Table 4:** Associations between caregiver knowledge and strategy use and child social-emotional and mental health outcomes

	**Changes in Child Emotion Regulation (Pre − 2-Mo)**				**Changes in Child Empathy (Pre − 2-Mo)**				**Changes in Child Sadness Over Wrongdoing (Pre − 2-Mo)**				**Changes in Child Mental Health Challenges (Pre − 2-Mo)**			
	*b*	*SE*	Lower 2.5% CI	Upper 2.5% CI	*b*	*SE*	Lower 2.5% CI	Upper 2.5% CI	*b*	*SE*	Lower 2.5% CI	Upper 2.5% CI	*b*	*SE*	Lower 2.5% CI	Upper 2.5% CI
Changes in Knowledge (Pre − Post)	**.55**	**.25**	**.003**	**.976**	− .40	.39	− 1.113	.378	− .42	.37	− 1.121	.348	.11	.43	− .741	1.102
Strategy Use (Post)	**.60**	**.20**	**.154**	**.893**	**.60**	**.22**	**.168**	**.916**	**.47**	**.28**	**.027**	**1.102**	− .05	.33	− .762	.430
Changes in Knowledge x Strategy Use	**.69**	**.29**	**.039**	**1.166**	− .19	.42	–.926	.769	− .37	.44	− 1.287	.442	− .19	.53	− .977	1.111

Bolded effects were statistically significant according to 95% bootstrapped confidence intervals with 5000 resampled draws

There were significant main effects of caregiver use of training strategies on changes in child empathy and sadness over wrongdoing, *b* = 0.60, 95% CI [0.168, 0.916] and *b* = 0.47, 95% CI [0.027, 1.102], respectively, such that caregivers who reported more frequent strategy use at post-training also indicated that their children showed steeper improvements in empathy and sadness over wrongdoing from pre-training to the 2-month follow-up. There were no associations between changes in knowledge, strategy use, or their interaction and changes in child mental health challenges.

### Research question 4

**Fig. 5:**
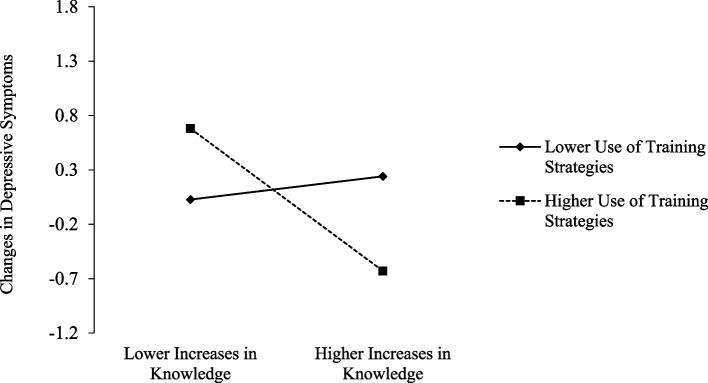
Interaction plot. Increases in knowledge from pre- to post-training in relation to changes in caregiver and educator depressive symptoms from pre- to 2-month post-training at lower (− 1 SD) and higher (+ 1 SD) levels of use of training strategies post-training. The observed range of changes in caregiver and educator depressive symptoms was − 2.00 to 2.00. Positive scores on changes in depressive symptoms denote increases, whereas negative scores denote decreases

As seen in Table 5, a significant main effect of changes in caregiver and educator knowledge on changes in caregiver and educator depressive symptoms, *b* =  − 0.34, 95% CI [− 0.750, − 0.012], was qualified by a significant interaction, *b* =  − 0.54, 95% CI [− 0.978, − 0.007], such that increases in participants’ training knowledge were associated with decreases in their depressive symptoms when use of training strategies was higher, *b* =  − 0.81, 95% CI [− 1.552, − 0.193]. This association was nonsignificant when use of training strategies was lower, *b* = 0.13, 95% CI [− 0.271, 0.425] (see Fig. 5). There was a significant main effect of caregiver and educator use of training strategies on changes in caregiver and educator anxiety, *b* =  − 0.35, 95% CI [− 0.643, − 0.067], such that those who reported more frequent strategy use also reported steeper declines in anxiety symptoms from pre-training to the 2-month follow-up. Wald tests revealed there were no significant differences between caregivers and educators on any effects (*ps* > 0.05), suggesting that the pattern results were consistent for caregivers and educators.

**Table 5:** Associations between caregiver and educator knowledge and strategy use and their depressive and anxiety symptoms

	**Changes in Caregiver and Educator Depressive Symptoms (Pre − 2-Mo)**				**Changes in Caregiver and Educator Anxiety Symptoms (Pre–2-Mo)**			
	*b*	*SE*	Lower 2.5% CI	Upper 2.5% CI	*b*	*SE*	Lower 2.5% CI	Upper 2.5% CI
Changes in Knowledge (Pre − Post)	− **.34**	**.18**	− **.750**	− **.012**	− .09	.23	− .454	.454
Strategy Use (Post)	− .07	.16	− .507	.173	− **.35**	**.15**	− **.643**	− **.067**
Changes in Knowledge x Strategy Use	− **.54**	**.24**	− **.978**	− **.007**	− .40	.27	− .840	.315

Bolded effects reflect statistically significant effects according to the 95% confidence interval results. Bootstrapping was conducted with 5000 resampled draws

## Discussion

This pilot study evaluated the feasibility of a brief social-emotional training for caregivers and educators of children ages 3 to 8 years, and showed signs of positive efficacy across all objectives. Notably, caregivers and educators showed adequate retention throughout the training and expressed high satisfaction with the initiative. Further, preliminary evidence revealed that both caregivers and educators reported significant increases in knowledge of social-emotional concepts that were maintained 2 months after the training. Additionally, the pilot evidence suggests that increases in participants’ knowledge and their greater use of the training-based strategies were associated with improvements in children’s social-emotional capacities and in their own mental health. These preliminary results suggest that the current training may be a fruitful response to existing gaps in caregiver and educator social-emotional training opportunities, and extend the existing literature by showing positive efficacy of a brief, research-based training initiative that aims to directly translate and apply social-emotional research and theory into accessible training for caregivers and educators.

The training showed preliminary evidence of being successful in improving both caregivers’ and educators’ knowledge of children’s core social-emotional concepts, such as emotion regulation, empathy, self-reflection, and sadness over wrongdoing. Specifically, caregivers’ and educators’ knowledge increased from pre- to post-training and newfound knowledge was maintained at the 2-month follow-up, suggesting some evidence of training sustainability. The RAISE training adopts a flexible, blended training structure whereby participants are given the autonomy to engage with core knowledge-based components of each module in a self-paced manner according to their own schedules and needs. These sessions were supplemented with weekly synchronous virtual sessions that emphasized group discussion, reflection, and application of core concepts to support deeper understanding of the content and translation into real-world practice. Participants reported high levels of satisfaction with the training and after each session, suggesting that they found value in the asynchronous and synchronous components, corroborating past work on the benefits of blended learning [59].

Increases in caregiver knowledge and greater use of training-based strategies were associated with steeper improvements in child social-emotional capacities in unique ways. Specifically, caregivers’ higher use of training-based strategies was associated with increases in their children’s empathy and sadness over wrongdoing. Further, caregivers’ increases in knowledge were associated with increases in child emotion regulation when they also reported greater use of training-based strategies. These findings add promising evidence to literature underscoring the utility of social-emotional training initiatives for supporting child outcomes [31]. They also align with ecological systems, family systems theory, and relational-developmental systems approaches whereby positive changes in children’s rearing environment are expected to cascade into positive changes in their development [68]. Intervening across children’s broader micro-system via caregivers’ and educators’ capacities to support child social-emotional development may be a fruitful approach to comprehensive and sustained developmental care.

Despite positive impacts on children’s social-emotional capacities, there were no preliminary associations between training outcomes and child mental health challenges. Notably, some theoretical and conceptual approaches identify emotion regulation (which was shown to increase with training outcomes) as a core feature of mental health [69]. Further, although the pilot training included a focus on the impacts of stress and mental health, it is possible that such impacts may occur further downstream through other effects of the training. In line with a developmental cascade perspective, impacts of the social-emotional training on child mental health may occur over a more protracted period through impacts on child social-emotional capacities [70]. Such effects may also occur through changes in caregivers’ and educators’ own mental health. Past longitudinal work evaluating long-term effects of social-emotional and related training initiatives has indicated evidence of “sleeper effects”, whereby training leads to delayed mental health benefits (e.g., later reductions in conduct problems or depressive symptoms) [71, 72]. For example, Jones and colleagues showed that effects of their social-emotional training led to reductions in child aggression and increases in social competence over a period of 2 years after the training occurred [73]. Our future work scaling up this intervention will aim to employ more extended longitudinal follow-ups to elucidate potential long-term or cascading effects of caregiver and educator social-emotional training on child mental health.

The current training also showed preliminary evidence of providing direct benefits for caregivers and educators. Specifically, caregivers’ and educators’ greater use of training strategies was associated with reductions in their depressive symptoms from pre-training to post-training. This might suggest that the training strategies alone may buffer feelings of hopelessness that are common with depression, perhaps because these strategies provide concrete examples of behaviors that can help support mental health. Further, caregivers’ and educators’ increases in knowledge were associated with reductions in anxiety when they also reported higher use of training-based strategies. Thus, it was the combination of high strategy use *and* increases in knowledge that was associated with reductions in anxiety. This might suggest that to reduce anxiety, it may be necessary to support adults’ recognition of the underlying development and rationale behind different strategies. It is possible that increases in knowledge work to facilitate increased confidence or feelings of control in their caregiving abilities, which, when coupled with higher strategy use, is accompanied by reductions in anxiety. Incorporating mental health strategies focused on supporting caregiver and educator mental health addresses gaps around adult well-being in existing social-emotional training initiatives, underscoring the importance of incorporating strategies (e.g., mindfulness, stress management, emotion regulation strategies) that caregivers and educators can use to support their own mental health and social-emotional capacities. Although the current findings require further replication, they may be promising given existing literature establishes deleterious links between caregiver mental health and child well-being [74]. Particularly, caregivers with psychopathologies such as depression are less likely to engage in positive parenting behaviors, which in turn pose risk for children’s well-being [75]. Thus, intervention approaches that aim to improve caregiver mental health symptoms *and* support their parenting behaviors may be particularly useful for supporting improved caregiver and child outcomes [76]. It is also possible that our approach of supporting caregivers and educators with research-based knowledge in a manner that recognizes and builds upon their existing competencies and strengths indirectly contributed to their mental well-being by supporting their sense of competence as caregivers and educators [77]. Relatedly, these results may also be explained by the theoretical and empirical notion that “helping yourself helps others”; in other words, increasing one’s capacity to tolerate their own distress and challenges may better equip them to show compassion and support for others [22, 78].

Our pilot results also highlight the importance of targeting both knowledge *and* practical application in social-emotional training initiatives. Knowledge of core social-emotional concepts and use of social-emotional promotion strategies supported child, caregiver, and educator outcomes. Further, associations between increases in providers’ knowledge and improvements in child emotion regulation and improvements in caregiver and educator anxiety symptoms were only significant when caregivers and educators *also* reported higher use of strategies. Thus, our results emphasize that it may not be enough to simply increase knowledge or use of the strategies. Instead, it may be most beneficial when caregivers and educators practice evidence-based strategies with a solid understanding of the developmental processes and mechanisms that underly those strategies. One caregiver noted the helpfulness of making connections between theory and application in daily life: “The training was incredibly helpful and very easy to apply in day-to-day life. This is so crucial because I have been in numerous training sessions that give some great theory…but don’t connect easily in our day-to-day lives.” When caregivers and educators are supported with the tools needed to make connections between theory and practice, this may support their capacity to act with informed, intentional care, thereby increasing the chances that their behaviors translate into improvements in children’s and their own social-emotional capacities and mental health [32]. With the results from this pilot study in mind, our future scaled-up implementation will strive to incorporate additional strategies and opportunities for practical application.

In sum, the present results are promising and seem to be aligned with past evidence indicating the utility of brief interventions as a cost effective and proportionally (or possibly even more) successful prevention approach relative to more intensive, lengthy, and costly intervention approaches [79]. Strengths of the current training include its strengths-based focus and its links with support positive changes at multiple levels of the child’s rearing environment. However, there are limitations to the present study that bear mentioning. First, only caregivers reported on children’s social-emotional capacities, which may have introduced shared method variance. Although caregiver reports can often provide informative and reliable insights into children’s well-being and social-emotional capacities [80], future related work should consider child self-reports or child behavioral assessments of social-emotional capacities and mental health. Our future work will evaluate changes in children under educators’ care as this can inform understanding of whether changes in educator knowledge and knowledge application translate to support child social-emotional and mental health outcomes in educational and child care settings. Finally, as a pilot study, the current project was limited in sample size. This precluded us from using more robust statistical techniques to assess training impacts, such as latent change and latent growth curve modelling. Similarly, the current study did not include a control group or a randomized controlled trial design, which prevented us from evaluating effects of the training relative to a between-person baseline. Participating caregivers and educators likely had some experience with the community services who supported recruitment for the current pilot, which may have impacted the potential generalizability of our results. Including further metrics of feasibility, such as proportion of the interested population that was reached, and formally reporting engagement in the live group sessions would have strengthened the current design. Clinical implications will become more apparent when the training is evaluated within a large-scale randomized controlled trial. Nonetheless, this feasibility study serves as a critical first step that informs our preparation for a full-scale randomized controlled trial of the present training.

## Conclusions

In sum, the current findings offer promising preliminary evidence for the efficacy of a novel research-based training initiative to support caregivers’ and educators’ capacities to nurture child social-emotional development. They emphasize the importance of infusing both research-based knowledge and practical strategy-based application into caregiver and educator training initiatives, and speak to the viability of targeting developmental change in children at the level of the caregiver. Further, they underscore caregivers’ and educators’ own mental well-being amidst the stress of home life and service settings as foundational to supporting positive child developmental outcomes.

## Data Availability

Due to the nature of this research, participants of this study did not agree for their data to be shared publicly, so supporting data is not available.

## Footnotes

1 Caregivers’ and educators’ responses after each asynchronous and synchronous training session were averaged into a single post-training score.

2 Internal consistencies of the original 10-item internalizing and externalizing scales were poor (*α* < .60). To address this, two factor analyses were conducted. Based on the factor analyses results, three items were removed from the internalizing scale (i.e., “My child is rather solitary, or prefers to play alone”, “My child has at least one good friend”, “My child is generally liked by other children”) and three items were removed from the externalizing scale (i.e., “My child is constantly fidgeting or squirming”, “My child is often argumentative with adults/lies or cheats”, “My child can be spiteful to others/steals from home or school or elsewhere”) to maximize internal consistency. Removing these items improved alpha to > .60 at all time points.
